# Destroyed Lung Syndrome in a Young Indian Male: A Case Report

**DOI:** 10.7759/cureus.38174

**Published:** 2023-04-26

**Authors:** Sankalp Yadav

**Affiliations:** 1 Medicine, Shri Madan Lal Khurana Chest Clinic, New Delhi, IND

**Keywords:** pneumonectomy, bronchiectasis, pulmonary aspergillosis, tuberculosis, destroyed lung

## Abstract

A destroyed lung pertains to the complete destruction of the lung. This is an irreversible condition and is an outcome of chronic or recurrent lung infections. Tuberculosis is widely reported to be a cause of destroyed lungs, and post-tubercular destroyed lung syndrome is a major issue, especially in countries with a high burden of tuberculosis. Herein, a case of destroyed lung syndrome in a 22-year-old Indian male is presented. He had a history of irregular treatment for tuberculosis and reported complaints of dry cough, fever, and dyspnea. A detailed clinical, radiological, and lab workup established the diagnosis of destroyed lung syndrome, and he was reinitiated on anti-tubercular treatment.

## Introduction

Tuberculosis continues to be a significant public health issue [1]. The disease is the major contributor to morbidity and mortality in endemic countries [2]. Per the WHO Global TB Report of 2022, there was an alarming rise in cases of tuberculosis in the year 2021 as compared to the previous year [2]. There were 134 cases per 0.1 million population and a total of 10.6 million cases in the year 2021 [2].

Treatment of tuberculosis is usually through well-established national programs [3]. These programs are monitored by the WHO, and emphasis is laid on making these services patient friendly [3]. However, post-treatment complications due to lung damage caused by the disease have remained unheeded. There is a paucity of data related to the complications post anti-tubercular treatment [4,5]. Some of these complications involve dyspnea either at rest or on exertion, recurrent hemoptysis, and persistent chest pain [6]. These complications ultimately affect the quality of life of tuberculosis survivors [6].

A case of a 22-year-old Indian male is presented. He had reported complaints of dry cough, fever, and dyspnea at exertion. Considering the history where he had taken anti-tubercular treatment for two months with a gap of more than one month, a detailed clinical, radiological, and lab workup was done which established the diagnosis of destroyed lung syndrome with tuberculosis, and he was reinitiated on anti-tubercular treatment.

## Case presentation

A 22-year-old Indian male belonging to a low-income family came with chief complaints of dry cough, fever, and dyspnea. The cough was intermittent without expectoration and was present for nearly three months. There was no hemoptysis, and his cough was relieved temporarily after taking over-the-counter antitussive syrup dextromethorphan. He had a fever that was insidious in onset evening rise and was not associated with chills or rigor for two weeks and was relieved after taking paracetamol. Furthermore, he had dyspnea at rest which was progressive over a period of two weeks. Initially, he had grade 1 dyspnea per the modified Medical Research Council dyspnea scale which progressed to grade 5 over a period of six months. There was no history of night sweats, loss of appetite, or weight loss.

He was diagnosed with drug-sensitive microbiologically confirmed pulmonary tuberculosis (sputum for the acid-fast bacilli (AFB) test was 1+) one year ago for which he was advised anti-tubercular treatment but he discontinued the treatment after two months as he started to feel better and, therefore, was declared as lost-to-follow-up. There was no history of any other major medical or surgical intervention in the past. There was no history of tuberculosis in the family or his contacts. Besides, there was no history of any similar complaints in close contacts, and there was no history of smoking, substance abuse, or exposure to silica.

A general examination revealed a cachectic man with a weight of 48 kilograms (BMI 16.8 kg/m^2^). He was afebrile to touch with a pulse of 88/minute, arterial blood pressure was 126/86 mm of Hg, respiratory rate was 29 breaths/minute, and oxygen saturation (SpO2) was 94% on room air. His SpO2 fell by 89% on room air after walking. There was no pallor, icterus, cyanosis, lymphadenopathy, or edema.

Systemic examination was remarkable for cavernous/amphoric breathing on the left side of the chest and vesicular breath sounds on the right side. Crepitations were heard over the entire left lung. The rest of the systemic examinations were within normal limits.

A preliminary diagnosis of tuberculosis was made, and he was referred to the lab for his sputum for an AFB test, a chest radiograph, and a cartridge-based nucleic acid amplification test of the induced sputum, with other routine investigations.

**Figure 1:**
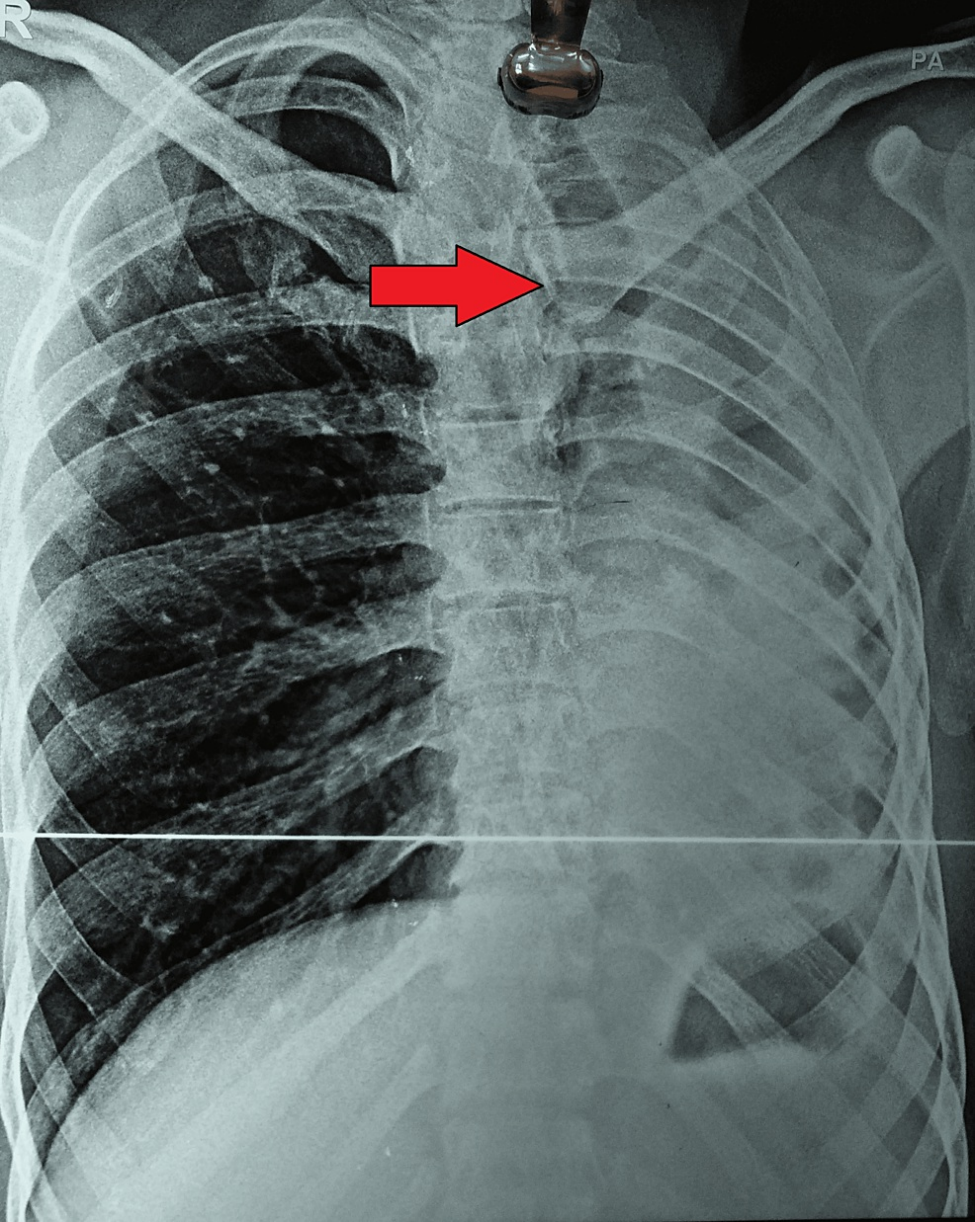
Chest radiograph (posteroanterior) view showing left lung involvement

The results of sputum for the AFB test and cartridge-based nucleic acid amplification test of the induced sputum were negative. However, the chest radiograph was suggestive of left lung collapse with a shift of upper and lower mediastinum toward the left side with decreased intercostal spaces (Figure 1).

**Figure 2:**
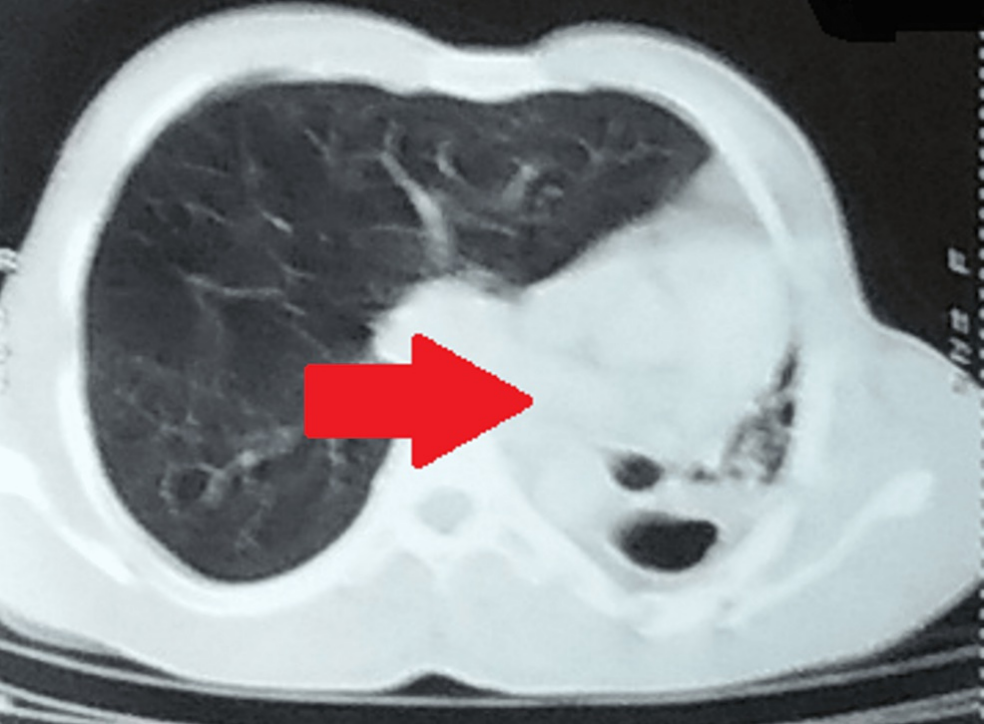
CT chest showing the collapse of the left lung

These findings were confirmed on CT of the chest which was remarkable for the collapse of the left lung with volume loss on the same side and ipsilateral crowding of the ribs with a mediastinal shift on the left side. Furthermore, fibrocystic changes with patchy consolidation and thick-walled cavitary lesions were seen in the collapsed left lung with decreased intercostal spaces. Compensatory over-inflation of the right lung was seen (Figure 2).

Pulmonary function tests were suggestive of obstructive disease with forced expiratory volume in 1 second (FEV1): 0.89 liters, forced vital capacity (FVC)-1.72 liters, and FEV1/FVC-57%. The rest of the laboratory workup was remarkable for mild anemia with hemoglobin of 11 g/dL. Furthermore, the sweat chloride test, aspergillus precipitin test, AFB-*Mycobacterium* other than tuberculosis (MOTT) blood panel test, the urine (routine/microscopic), and thyroid profile were normal, and his HIV status was non-reactive. The sputum bacterial culture was sterile.

Finally, a diagnosis of pulmonary tuberculosis with tuberculous destroyed lung was made, and he was initiated on anti-tubercular treatment with four drugs, i.e., rifampicin, pyrazinamide, isoniazid, and ethambutol, as a fixed-dose combination per the National Tuberculosis Elimination Program. For his dyspnea, he was prescribed a dry powder inhaler of salmeterol plus fluticasone 250 microgram twice daily, a dry powder inhaler of tiotropium 18 microgram once daily, a tablet of acebrophylline 200 mg at bedtime daily, breathing exercises with incentive spirometry, and yoga. Dietary advice included a high-protein diet and regular walking for maximum tolerance. He was also advised to take influenza vaccination annually and the pneumococcal vaccine every five years. The patient was counseled for treatment adherence and regular follow-up at the nearest health center and was transferred out to his native village at his request. Furthermore, from the first episode of tuberculosis till the diagnosis of pulmonary tuberculosis with tuberculous destroyed lung, a total duration of one year was noted in this case.

## Discussion

Tuberculosis is a major cause of concern for healthcare systems in high-burden countries of Asia and Africa [2]. The disease is treated through well-defined guidelines implemented through national programs like the National Tuberculosis Elimination Program of India [3]. However, a large population of post-treated cases ends up being symptomatic [6]. These symptoms are the outcomes of irreversible lung damage due to chronic infections [6]. One such complication is the destroyed lung which is defined as the complete or extensive destruction of the lung with a compromised lung function [7,8]. It is an irreversible condition with a blend of pleural and parenchymal lung dismantling with cavitation, bronchiectasis, loss of lung volume, and mediastinal herniation to the ailing side [9]. Radiological studies help in establishing the diagnosis and are characterized by a remarkably reduced ventilation-to-perfusion ratio [10]. Tuberculosis is reported as the commonest cause of a destroyed lung, and it could happen after a primary disease or reinfection [4,11]. However, there is a paucity of literature related to it, and there is no exact prevalence or incidence known. Other causes include cystic bronchiectasis, aspergillosis, emphysema, multiple or extensive lung abscesses, lung gangrene, necrotizing pneumonia, and mycobacteria other than tuberculosis [10,12].

The components of destroyed lung syndrome include pulmonary cavitation, cystic bronchiectasis, loss of lung volume, pleuroparenchymal fibrosis, unilateral near complete lung parenchymal anomalies with combinations of the above findings, contralateral lung parenchymal compensatory hyperinflation manifested as emphysema, and pull of contralateral lung and mediastinal structures to diseased side radiologicalLy termed as mediastinal herniation [9]. A reduction of intercostal spaces resulting in the crowding of ipsilateral ribs is also seen.

The clinical presentations are mostly chronic, but acute cases are also documented [13]. The patient may present with prolonged and frequent episodes of respiratory illnesses, purulent (yellow-colored) expectoration, chronic low-grade fever, dyspnea, chest tightness, and generalized ill health and may be complicated with episodes of recurrent hemoptysis [4,11,13]. Acute symptoms of septicemia, massive hemoptysis, or respiratory failure may also be present [11].

In a study by Dhar et al. on 2195 study subjects, it was reported that post-tuberculosis lung damage led to a loss of nearly 40% of lung capacity in study subjects [14]. This ultimately results in dyspnea and adversely affected the quality of life [14]. In another prospective cohort study from Malawi on 405 study subjects, Meghi et al. inferred that post-tuberculosis lung damage was common and under-recognized [15]. Post successful completion of anti-tubercular treatment, this lung damage is incapacitating for patients and linked with adverse outcomes beyond completion of anti-tubercular treatment [15]. In another study by Hossain et al. from Bangladesh on 600 study subjects, nearly 70% had destruction of the left lung [11]. Furthermore, tuberculosis was attributed as the basis of lung destruction in 84% of the study subjects [11].

A case similar to the present case was published by George MD in 2013, where a 45-year-old man was diagnosed with destroyed lung syndrome [16]. The present case shares similarities with that case with features a history of tuberculosis, dyspnea, and radiological findings like mediastinal shift, destroyed lung, and hyperinflation of the contralateral lung [16]. However, the present case differs from their case in the side which was the left lung, age, ethnicity, presence of dry cough and fever, absence of hemoptysis, dextrocardia, and hydropneumothorax [16].

Another case series similar to the present case was published by Patil et al., where 42-year-old and 49-year-old male patients were diagnosed with destroyed lung syndrome post-tuberculosis sequel [9]. The present case shares similarities with their two cases in radiological findings, gender, ethnicity, and symptoms of cough, fever, and dyspnea [9]. However, the present case differs from both the cases in the side which was the left lung as compared to the right lung in their cases, with the absence of expectoration and sterile culture with no growth of any microorganism [9]. Besides, similar to their second case, the present case has a history of incomplete anti-tubercular treatment.

Histopathologically, extensive fibrosis as seen in the present case is usually seen in destroyed lung syndrome [13]. Besides, parenchymal destruction is more if the pathological involvement of the left lung is present [13]. This is attributed to the anatomy and the layout of the left bronchus which is narrower, longer, and comparatively more horizontal ultimately affecting the drainage of secretions [13]. In the present case, the left hemithorax is involved and radiological findings in the thoracic region (e.g., mediastinal shift, decreased intercostal spaces, retraction of the affected hemithorax) of the presented patient were quintessential of destroyed lung syndrome and were suggestive of a chronic process.

Moreover, there are no definite treatment guidelines available [4]. Conservative management involves symptomatic treatment with the use of long-acting muscarinic antagonists or long-acting beta-2 agonists plus inhaled corticosteroids leading to bronchodilatory effects [17]. Yum et al. mentioned that tiotropium had a therapeutic effect on tuberculous destroyed lungs [18]. In a recent multi-center, double-blind clinical trial, indacaterol was divulged to significantly increase FEV1 and ameliorated dyspnea compared with placebo [19]. Surgical management involves a high-risk procedure, i.e., pneumonectomy, which is indicated in the management of destroyed lungs to either resolve or avert complications [4,8].

Extensive tuberculous lung destruction-related mortality is high and is mostly associated with massive hemoptysis, superinfections, respiratory failure, or the presence of active tuberculosis [20]. Furthermore, hemoptysis due to hypertrophied bronchial arteries or Rasmussen aneurysms could result in lethal complications in cases with tuberculous destroyed lung syndrome [11].

Furthermore, severe complications, such as septicemia/empyema and left-right shunt, are also reported in cases of destroyed lung syndrome [13]. These shunts could result in pulmonary hypertension and respiratory failure despite having a normal contralateral lung [13].

It is imperative that supervision and monitoring should be strict to prevent any lost-to-follow-up cases. Regular counseling of patients on anti-tubercular medicines is essential, as high pill burden, long course of treatment, and adverse drug reactions often result in incomplete treatment which ultimately could end up with destroyed lung syndrome.

## Conclusions

Tuberculosis is rampant in endemic countries. The cases of tuberculous-destroyed lung syndrome are alarming as less attention is paid to the patient’s post-completion of anti-tubercular treatment, and this often impacts the quality of life of tuberculosis survivors. The present case will help create awareness about this condition which, if went unheeded, could end up with complications like massive hemoptysis, respiratory failure, superinfections, or reactivation of tuberculosis. Although the present case was reported to the healthcare facility after a gap of nearly 10 months, this case highlights the problem of managing such cases in countries where the burden of disease is very high with no clear guidelines for retrieving and managing such post-tubercular cases with destroyed lung syndrome. Therefore, this case would help clinicians keep an eye on such presentations and would help them manage such cases.
